# Vegetation greenness modelling in response to interannual precipitation and temperature changes between 2001 and 2012 in Liao River Basin in Jilin Province, China

**DOI:** 10.1186/s40064-016-2737-9

**Published:** 2016-07-26

**Authors:** Xiao-sheng Lin, Jie Tang, Zhao-yang Li, Hai-yi Li

**Affiliations:** 1 Key Lab of Groundwater Resources and Environment, Ministry of Education, Jilin University, 2699 QianJin Street, Changchun, 130012 Jilin People’s Republic of China; 2 College of Environment and Resource, Jilin University, 2699 QianJin Street, Changchun, 130012 Jilin People’s Republic of China

**Keywords:** Normalized Difference Vegetation Index (NDVI), Vegetation greenness, Climatic variability, Correlation analysis

## Abstract

**Background:**

Liao River basin in Jilin Province is the place of origin of the Dongliao River. This study gives a comprehensive analysis of the vegetation coverage in the region and provides a potential theoretical basis for ecological restoration.

**Methods:**

The seasonal variation of vegetation greenness and dynamics based on the Normalized Difference Vegetation Index (NDVI) in major land cover types in the region was studied. Analyzing the relationship NDVI, temperature and rainfall, we derived a set of predictor variables from 2001 to 2012 using the MODIS Terra level 1 Product (MOD02QKM).

**Results:**

The results showed a general increasing trend in NDVI value in the region, while 34.63 % of the region showed degradation. NDVI values begin to rise from April when plants are regreening and they drop in September when temperature are decreasing and the leaves are falling in the study area and temperature was found decreasing during the period of 2001–2012 while rainfall showed an increasing trend. This model could be used to observe the change in vegetation greenness and the dynamic effects of temperature and rainfall.

**Conclusion:**

This study provided important data for the environmental protection of the basin area. And we hope to provide scientific analysis for controlling water and soil erosion, maintaining the sustainable productivity of land resources, enhancing the treatment of water pollution and stimulating the virtuous cycle of the ecological system.

## Background

Vegetation, as the main body of terrestrial ecosystem, serves as a bearer as well as a feedback of climate change. It acts as an indicator in the study of global climate change (Liu and Ren 2012; Cui et al. 2009), which has greatly influenced terrestrial ecosystem (IPCC 2007; Zhao et al. 2011). Thus the study of vegetation’s spatiotemporal change and its response to climate change has always been an important component in the study of global climate change (Zhang et al. 2011; Xin et al. 2007). Up till now, more than 40 kinds of vegetation indices have been defined and widely applied to various studies ranging from the analysis of global and regional land coverage, vegetation classification and environmental changes (Guo et al. 2002), primary productivity analysis (Sun and Zhu 2000; Xiao et al. 1996), potential yield of crop and pasture assessing (Xiao et al. 1986) to drought monitoring (Chen et al. 1994; Guo et al. 1997a, b). The Normalized Difference Vegetation Index (NDVI) is widely used index of vegetation growth and vegetation coverage (Du et al. 2008) and the remote sensed satellite imagery allowed one to calculate the NDVI (Tucker 1979). It’s a commonly used remote sensing vegetation index in climate-phenology studies (Myneni et al. 1997; Zhou et al. 2001; White et al. 1997; Reed et al. 1994; Stockli and Vidale 2004) and calculated from the reflectance in the red and near infrared (NIR) bands of the electromagnetic spectrum and is a measure of the photosynthetic activity within the area covered by a pixel (Justice et al. 1985; Tucker and Sellers 1986). We use the Moderate Resolution Imaging Spectroradiometer (MODIS) sensor onboard the United States National Aeronautics and Space Administration’s (NASA) Terra spacecraft to monitoring vegetation changes in the whole region. The MODIS NDVI is considered to be an improvement over the NDVI product derived from the AVHRR sensors (Huete et al. 2002; Mildrexler et al. 2009; Peckham et al. 2008; Wang et al. 2006). NDVI have been used for ecological studies to determine overall productivity, biomass and seasonal variability in productivity and phenology and many studies dedicated to vegetation greenness response to climate variables by using NDVI (Guerschman and Paruelo 2005; Wang et al. 2008; Mao et al. 2012). A vegetation greenness model was developed from correlations between NDVI and meteorological data using the linear regression in Northeast Thailand (Watinee and Netnapid 2013). In Eastern China, the correlation coefficients of NDVI with temperature is larger than those with precipitation (Cui et al. 2010). This conclusion was further confirmed by research in Inner Mongolia of China (Shi et al. 2011). In addition, lag-time effects have been shown in related studies, precipitation and temperature changes often precede vegetation changes, with the time lag exhibiting some regional dependence (Potter and Brooks 1998; Richard and Poeeord 1998). The presence of lag-time effects was further confirmed on the prairies of Kansas and northern America (Wang et al. 2003). However, few works have been done on the analysis of the temporal and spatial patterns of vegetation dynamics in Liao River Basin, China. Previous findings and the current research mentioned above led to our research questions: How has the level of greenness observed in different land use type in this river basin changed over the past decade? What are the regional patterns of change in the vegetation dynamic (NDVI variation)? How do the effects of temperature and precipitation on vegetation dynamics differ spatially? The objectives of this study were (1) to analyse NDVI, precipitation and temperature changes during the past 10 years in Liao River Basin; (2) to compare correlations between NDVI, Temperature and precipitation in the spatial scales; (3) to asses whether climatic variables can be used to identify changes in vegetation in the Liao River Basin.

## Datasets and methods

### Study area

**Fig. 1:**
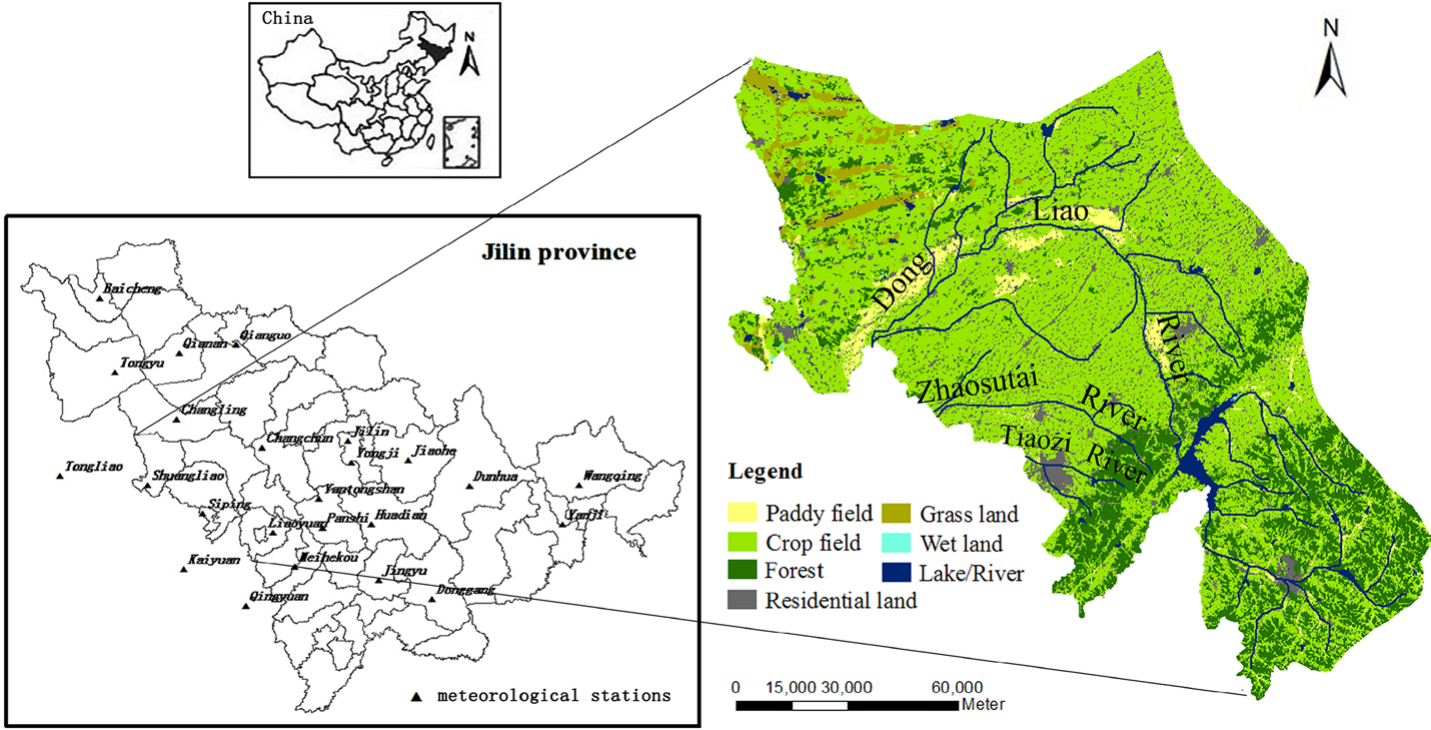
The study area and the locations of the meteorological stations

Liao river basin, located in the southwestern of Jilin province (E 123°18′–125°36′, N 42′36°–44′10°) (Fig. 1), is adjacent to Tieling city of Liaoning province and Zhelimu Meng in Inner Mongolia. With a total area of 15,746 km^2^, Liao River basin covers a land of 8 % in the above-mentioned provinces. Dongliao River and Zhaosutai River are the main rivers. The basin is located in the middle of Songliao plain, with an elevation of 611–120 m. Forest, crop field (mainly cultivated corn and soybean) and paddy field (mainly cultivated rice) are the main land use type. The basin belongs to the temperate continental monsoon climate zone. The annual rainfall and evaporation is 545 and 1020 mm, respectively.

### Datasets

#### Meteorological data

The meteorological data used for trend analysis included temperature and rainfall collected from the China Meteorological Data Sharing Service System for the period from 2001 to 2012. Annual temperature and rainfall variables for analysis included the maximum temperature (Maxtemp), the minimum temperature (Mintemp) and the mean temperature (Mtemp). The monthly data were digitally encoded into a GIS database and the attribute values were linked to meteorological stations located near the region. In order for more accuracy, a total of 24 meteorological stations were used. An ordinary kriging method was applied under ArcGIS10.0, and then grid maps were produced (Maribeth 2012) for annual precipitation and temperature covering the whole region.

### Annual NDVI

The NDVI data (from 2001 to 2012) were taken from MODIS Terra level 1 product. The data were obtained from the NASA web interface under the Level 1 and Atmosphere Archive and Distribution System (LAADS) (http://ladsweb.nascom.nasa.gov/). We derived a set of predictor variables from 2001 to 2012, and the data reflectance of the red and near-infrared channels with the resolution of satellite data is 250 m × 250 m. A 1:1,000,000 vegetation map of China (Hou 2011) was used to explore the vegetation changes in different biome types. Following the criteria for biome type classification, vegetation in the study area was categorized into five main types: paddy field, crop field, grass land, forest and wetland. The NDVI was proposed by Rouse et al. (1974) based on the differences in pigment absorption features in the red and near-infrared region of the electromagnetic spectrum (1–1). The values of NDVI range from −0.1 to 0.1, the increase of positive NDVI values indicates a larger amount of green vegetation. NDVI values near zero and decreasing negative values indicate non-vegetated features such as barren surface (soil or rock), snow, water and clouds (Schnur et al. 2010).

1 $$ NDVI = \frac{NIR - RED}{NIR + RED} $$

where RED is the spectral reflectance obtained in the visible zone, and NIR is that obtained near infrared regions.

### Trend analysis of NDVI

A simple linear regression model was used to calculate the spatial changing pattern of NDVI (Ma et al. 2006) in ArcGIS10.0 with time as the independent variable and NDVI as the dependent variable. The outputs of the trend analyses are maps of regression slope values. The calculation formula is:

2 $$ Slope = \frac{{n \times \sum\nolimits_{i = 1}^{n} i \times M_{NDVI,i} - \sum\nolimits_{i = 1}^{n} i \sum\nolimits_{i = 1}^{n} {M_{NDVI,i} } }}{{n \times \sum\nolimits_{i = 1}^{n} {i^{2} } - \left( {\sum\nolimits_{i = 1}^{n} i } \right)^{2} }} $$

where variable *i* stands for the serial number of year *i*, which indicates the average NDVI of year *i*. The formula reflects the changing trend of NDVI in the study area in 12 years.

### Correlation analysis between NDVI and climatic variables

The correlation analysis was used to calculated the spatial and time series correlation between NDVI and climatic variables (Raynald 2005):

3 $$ r_{xy} = \frac{{\sum\nolimits_{i = 1}^{n} {(X_{i} - \overline{X} )(Y_{i} - \overline{Y} )} }}{{\sqrt {\sum\nolimits_{i = 1}^{n} {(X_{i} - \bar{X})^{2} } } \sqrt {\sum\nolimits_{i = 1}^{n} {(Y_{i} - \overline{Y} )^{2} } } }} $$

where *r*_*xy*_ is the coefficient for the two samples, *X*_*i*_ and *Y*_*i*_ is sample sizes, and $\overline{X}$ and $\overline{Y}$ are the average indexes of the samples. The value of the coefficient should be taken in the range of [− 1, 1]. The bigger the value is, the larger is the correlation between each variable. And when the value comes most closely to 0, the variable indicate the least correlation.

The linear regression between NDVI and climate variables was derived from different land cover types, and used to evaluate the impact of climatic conditions to the vegetation. For each land cover type, the evaluation of climate-controlled vegetation greenness was carried out using a multiple linear regression method between NDVI and meteorological data from 2001 to 2012. In this study, the effectiveness of the model was measured from adjusted coefficient of determination (Adj.R^2^), standard error of the estimate (Std.Error) and level of significant, the closer the value of Adj.R^2^ comes to 1, the more precise the model proves to be. The formula is (Raynald 2005):

4 $$ R^{2} = \frac{{\sum {(\hat{y} - \bar{y}^{2} )} }}{{\sum {(y - \bar{y}^{2} )} }} $$

5 $$ Adj.R^{2} = 1 - (1 - R^{2} )\frac{n - 1}{n - k - 1} $$

where n − 1 is degree of freedom of the residual sum of squares, n − k−1 is degree of freedom of the total sum of squares, $\sum {(\hat{y} - \bar{y}^{2} )}$ is regression sum of squares, $\sum {(y - \bar{y}^{2} )}$ is total sum of squares.

NDVI is not immediately responsive to rainfall, but NDVI tends to be a delayed effect of rainfall by 1 or 2 months (Grist et al. 1997). Thus, rainfall in the concurrent month and accumulation with one or two prior months were included and examined to find the best correlation with NDVI.

## Results

### Annual changes in temperature and rainfall

**Fig. 2:**
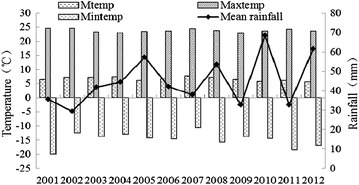
The annual temperature (°C) and rainfall (mm) time series

Figure 2 illustrates the changes in temperature and rainfall during 2001–2012. The max temperature is 24.57 °C in 2007 and the mintemp is −20 °C in 2001. The mean temperature (Mtemp) shows a declining trend which the value is −0.107 °C year^−1^ (R^2^ = 0.2876), respectively. The temporal change of rainfall in the region shows a positive trend in the period. With the peak values are 57.25 mm in 2005, 53.45 mm in 2008, 68.57 mm in 2010 and 61.64 mm in 2012; the lowest value is 29.49 mm in 2002.

**Fig. 3:**
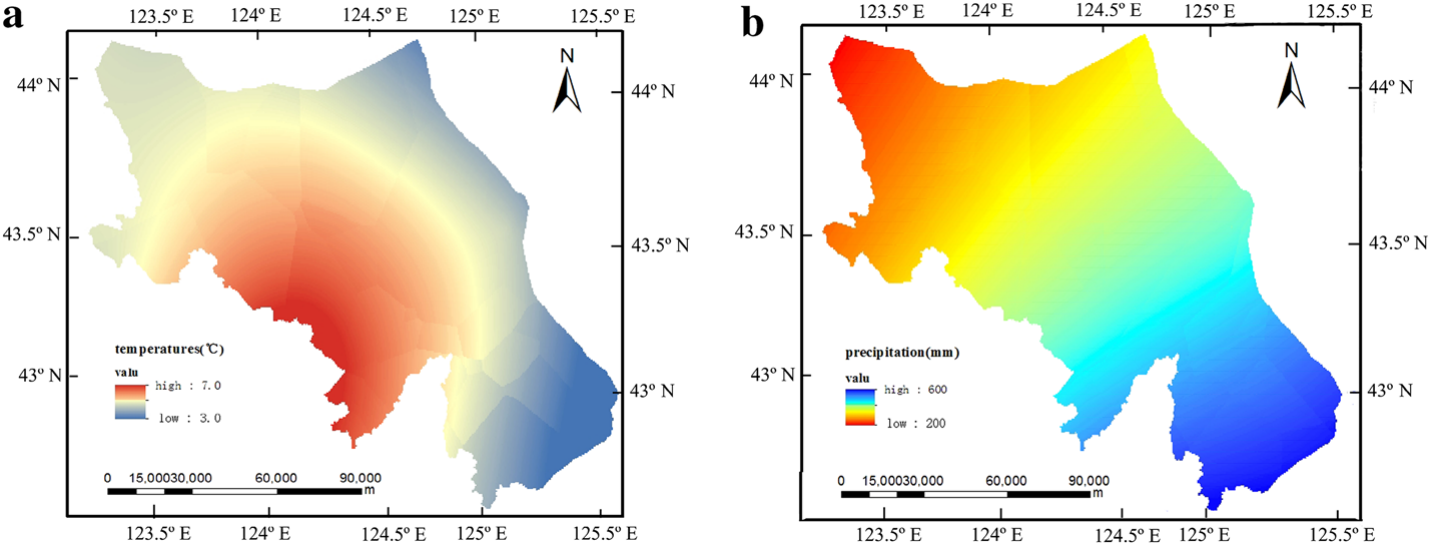
Spatial distribution of annual average temperature (°C) (**a**) and rainfall (mm) (**b**) in Liao river basin, China during the period of 2001–2012

Figure 3 shows that the spatial distribution of the annual average temperature (a) and rainfall (b) in 2001–2012.

From the figure, it is evident that the temperature is higher in Southwestern part of the region ranging from 6.4 to 7.23 °C, and the southeastern. The spatial distribution of annual rainfall shows that there is less rainfall in the Northwest of the region, ranging from 368.1 to 406.1 mm; and more rainfall is obtained in Southwest of the region with a range of 516.5–570.5 mm.

### NDVI changing patterns

**Fig. 4:**
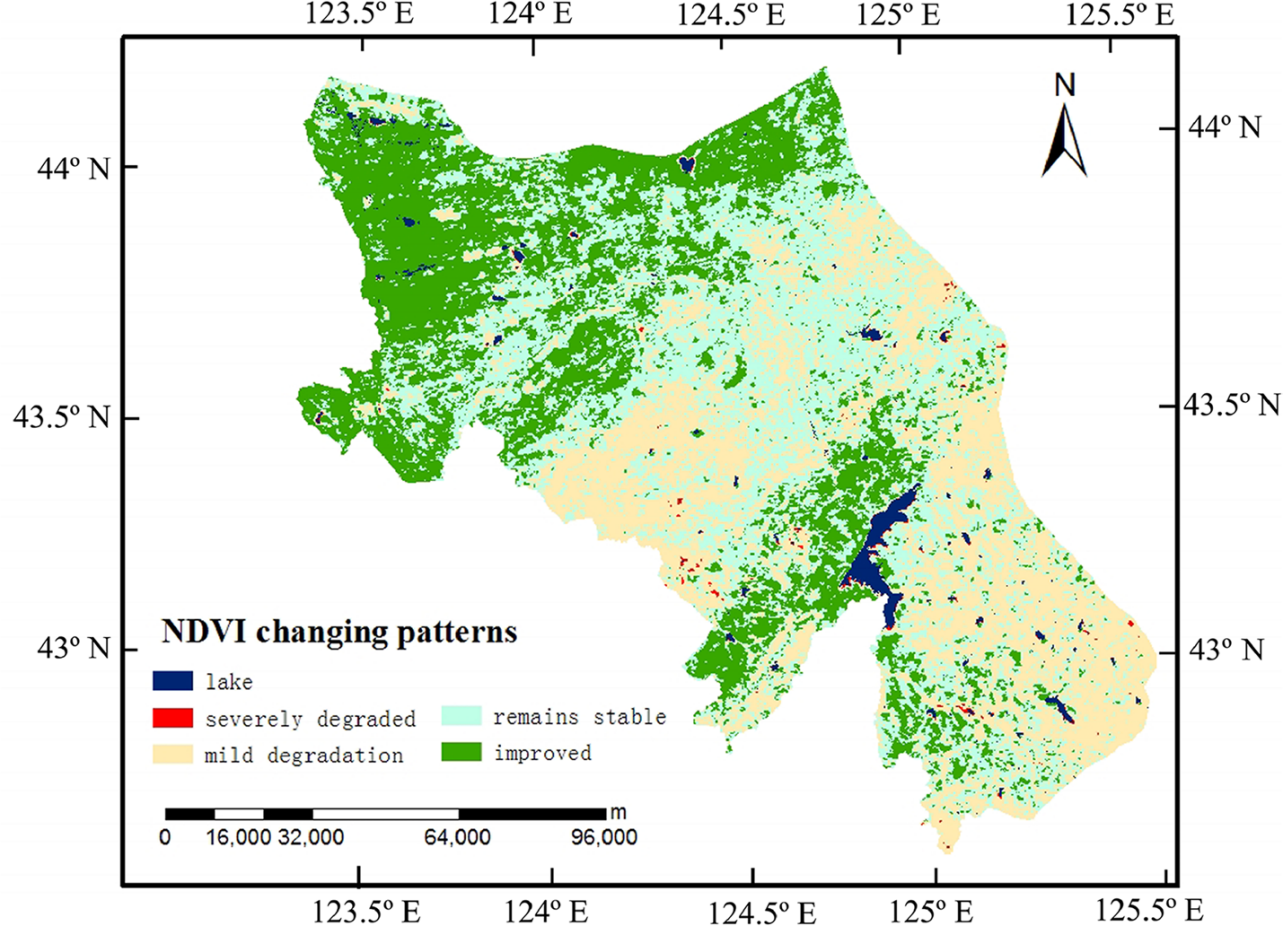
The linear trend regression slope values of NDVI (2000–2012 monthly data)

We used linear regression to analyze the change of NDVI during 2001–2012. The NDVI changing patterns are simulated based on the slope which is the gradient of the trend line. We classify the linear trend regression slope value as five levels: severely degraded (−0.05 to −0.01 year^−1^); remains stable (−0.01 to 0.01 year^−1^); mild degradation (0.01 to 0.05 year^−1^); improved (>0.05 year^−1^). The results are shown in Fig. 4.

**Fig. 5:**
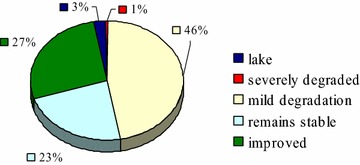
The proportion of pixel amounts for four areas with NDVI variation and lake area with no NDVI record

In 2001–2012, severely degraded vegetation in the region accounts for 3.13 % of the total area. 31.5 % of vegetation showed mild degradation. Most of the native land showed mild degradation, while 29.74 % were ameliorated in terms of vegetation coverage, stable vegetation coverage was observed in 32.52 % of the region. The proportion of pixel amounts for four areas with NDVI varied in the order of: mild degradation > improved > remain stable > severely degraded. Mild degradation account for nearly half of all pixels (Fig. 5).

**Fig. 6:**
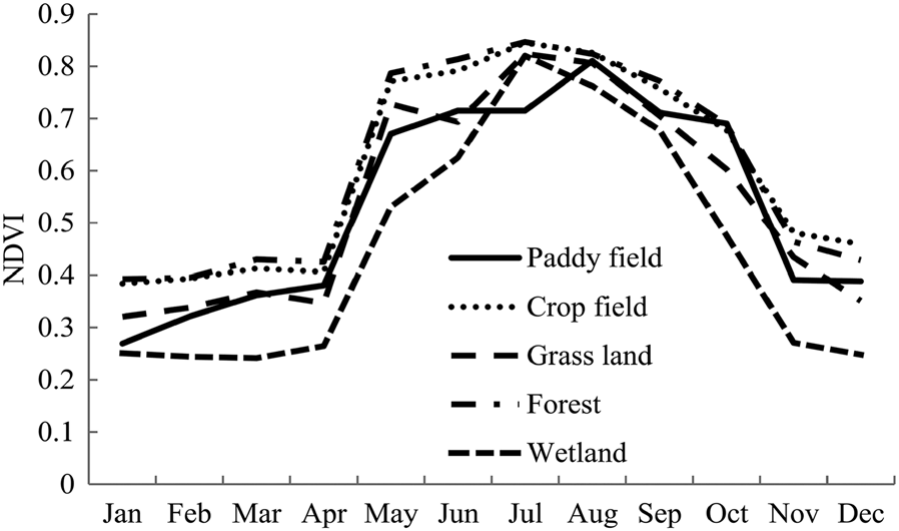
Monthly average NDVI for different land uses during the period of 2001–2012

The results show the monthly average NDVI for different land uses in the region during 2001–2012 (Fig. 6). The period from April to September was the growing season of the regional plants. NDVI values began to rise from April when plants were regreening and they dropped in September when temperature are decreasing and the leaves were falling. NDVI values remained stable in winter and early spring from November till the next March. Maximum NDVI value of Paddy field is perceived in August (0.81), while that of crop lands, grasslands, wetlands and forest were obtained in July, with 0.845, 0.824, 0.82, 0.847 respectively.

**Fig. 7:**
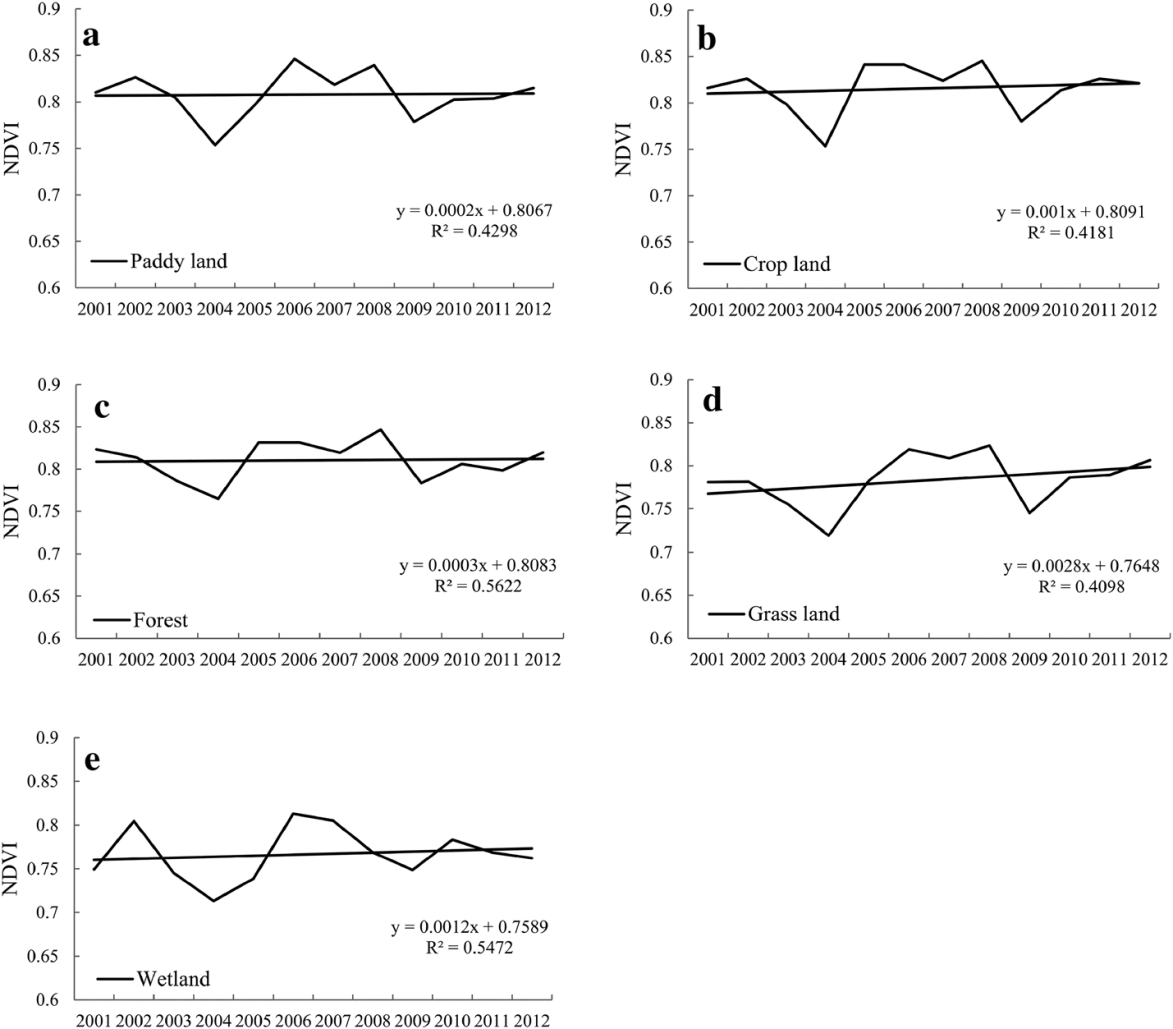
The annual changes in NDVI time series for different land uses during the period of 2001–2012. The land use types are: **a** paddy land, **b** crop land, **c** forest, **d** grass land, **e** wetland

The annual NDVI did not change significantly (Fig. 7). During the study period, different land use types of NDVI value fluctuated, with the year of 2004 exhibiting the lowest value. The peak value of the paddy fields and grassland were 0.852, 0.837 in 2006 and 0.848, 0.843 in 2008; Crop field and forest were 0.852 and 0.852 in 2008; wetland were 0.817 in 2002 and 0.822 in 2006.

### Correlations between NDVI, rainfall and temperature

**Fig. 8:**
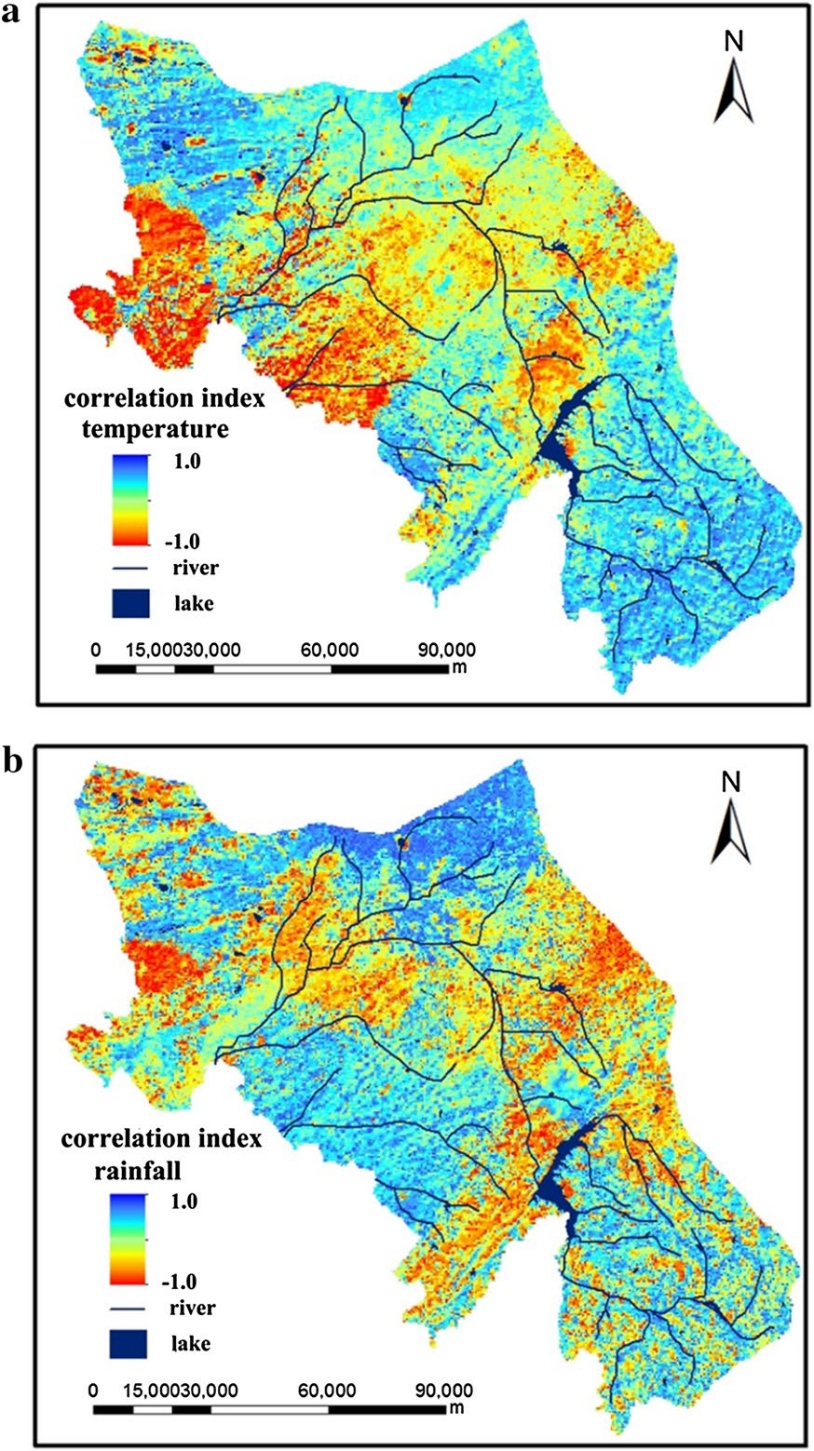
Spatial distribution of correlation between NDVI and climate factors. **a** The correlation index of NDVI and temperature; **b** the correlation index of NDVI and rainfall

Spatial pattern of NDVI–climate relationships was undertaken using the binary multiply method, and the results are shown in Fig. 8.

Climate variation can lead to significant changes in NDVI. The NDVI of upstream is in positive correlation with temperature, and is largely affected by temperature. Rich in water resources, the region is covered mainly by forests; while the downstream area is in negative correlation with temperature with the main type land cover of paddy field. Therefore, vegetation is more sensitive to temperature changes. The percentage of pixels that NDVI were significantly positively correlated and significantly negatively correlated with temperature is 17 and 39 %, respectively. Correspondingly, that with rainfall is 36 and 23 %, respectively (Table 1). In Table 1, the percentage of pixels in the significance level of 0.05 excluded the percentage of pixels in the significance level of 0.01. The increase of temperature results in the decrease of NDVI and the NDVI is in inverse proportion with the precipitation level at mid-eastern part of the region. This is not difficult to understand, Erlong reservoir is located in this area, featuring with low temperature and relatively slower physiological activities like plant photosynthesis and so on. The increase of precipitation level plays a detrimental effect on vegetation growth.

**Table 1:** The percentage of pixels which NDVI were positively related and negatively related with air temperature and Rainfall in the significance level of 0.01 and 0.05

	Temperature (%)	Rainfall (%)
Positively correlated (p < 0.01)	6	12
Positively correlated (p < 0.05)	11	24
Negatively correlated (p < 0.01)	14	10
Negatively correlated (p < 0.05)	25	13

The correlation coefficients are represented in Table 2 for each land use type with rainfall and temperature.

**Table 2:** Correlation coefficients (r) for NDVI and climate variables, for different land use types

Land cover type	Maxtemp	Mintemp	MTemp	MeanRainfall	LogMeanRainfall
Paddy field	0.222	0.114	0.219	0.499**	0.499**
Crop field	0.158	0.045*	0.132	0.464*	0.461*
Forest	0.103	0.058	0.080	0.306*	0.299
Grass land	0.105	0.103*	0.075	0.449*	0.444*
Wetland	0.391*	0.138	0.324	0.176	0.195

** At the 0.01 level was significantly correlated

* At the 0.05 level was significantly correlated

NDVI for paddy fields are correlated to rainfall accumulation and the correlation coefficient is 0.499 (p < 0.001), it is also correlated to log rainfall accumulation with a correlation coefficient of 0.499 (p < 0.001). A log-linear correlation between NDVI and rainfall were analyzed because that the saturation level of NDVI should remain constant even when rainfall increases (Petja et al. 2004). NDVI for crop fields, forest and grass land are also high correlated to rainfall accumulation and the correlation coefficients are 0.464, 0.306 and 0.449 respectively. The wetland is most correlated with Maxtemp and the correlation coefficient is 0.391.

It is analyzed that the correlation between NDVI and rainfall of the same month, rainfall that have been accumulated for 1 and 2 months respectively. A comparison between the precipitation level and NDVI shows the fact that the precipitation level affected NDVI when rainfall was accumulated for 2 months, as shown in Table 3.

**Table 3:** The correlation coefficient of precipitation in different calculation periods

Different period	April	May	June	July	August	September	October
The same period	−0.350	−0.007	−0.112	0.231	0.14*	0.14*	0.224*
Delayed for 1 month	0.356	0.203	0	−0.182*	0.014	−0.35**	0.07
Delayed for 2 months	0.028	0.035	0.713**	0.51**	0.357	0.063	0.462

** At the 0.01 level was significantly correlated

* At the 0.05 level was significantly correlated

The analysis of the delayed effect of seasonal rainfalls to NDVI shows good correlation between NDVI in June, July and rainfall accumulated for two months, with the correlation coefficients of 0.713 and 0.51 respectively (p < 0.01).The precipitation process influences vegetation growth by decomposing soil organic matters, improving soil nutrients and moisture. It takes time for rainfall to sift into vegetation roots, which explains vegetation’s delayed effect to rainfall (Hou et al. 2012).

We got the most suitable model for the region through linear regression analysis. Stepwise regression approach was used to analyze the significance of independent variables and the variables that are not significant were removed from the equation. Table 4 shows the regression model of NDVI of different land types, rainfall and temperature.

**Table 4:** Regression models between NDVI and rainfall and temperature variables monthly for different land cover types

Land cover	Model	Adj.R^2^	Std.Error	*p* value
Paddy field	NDVI = 0.798 + 0.001 MeanRainfall + 0.002 logMeanRainfall	0.597	0.043	0.038
Crop field	NDVI = 0.807 + 0.01 MeanRainfall	0.495	0.025	0.000
Forest	NDVI = 0.801 + 0.026 MeanRainfall	0.387	0.021	0.001
Grass land	NDVI = 0.769 + 0.015 MeanRainfall	0.704	0.026	0.037
Wetland	NDVI = 0.765 + 0.013 MaxTemp	0.370	0.031	0.001

NDVI for paddy field was explained by positive correlation with MeanRainfall and logMeanRainfall. Crop field, forest and Grass land were only explained by Mean Rainfall, NDVI for wetland was only explained by MaxTemp. The regression model of NDVI for Grass land was found more significant with higher Adj.R^2^ of 0.704 compared with other land cover types.

The correlations NDVI, temperature and rainfall respectively for this study altered from the variation of vegetation cover types. There were positive correlation between NDVI and rainfall for paddy field and crop field because of the dependence of the vegetation coverage to water for agricultural activities during the crops´ growing period. Positive correlations are also observed between forests and rainfall because this forest type conserves water in summer for its growth. Grassland was found to be in positive correlation with rainfall, for the grasslands spread mostly at the northwestern part of the region where the temperature is higher and the rainfall is lower. Abundant rainfall can lead to significant changes in NDVI values. Temperature was found positively correlated with wetland because that increasing temperature provides better condition for the growth of wetland plants.

## Discussion

### Annual changes in NDVI, temperature and rainfall

Temperature has been observed in the region during the 12-year period (2001–2012). The annual temperature observed in the region decreased and lower temperature was found in the southeastern part of the region. Rainfall in the region increased during the 12-year period and less rainfall was found in the northwest part of the region.

In this study, the linear trend regression slope values of NDVI revealed that 3.13 % of the region is severely degraded, 31.5 % is mildly degraded, and 62.26 % remains stable or is improved in vegetation coverage, Single peak variations have been shown in the seasonal changes of NDVI in different regions. NDVI values demonstrate significant increase since April and showed the peak in July or August. The value for Paddy land reached its maximum in August, while other land types peaks in July. The maximum values are as following: Paddy field: 0.81, Crop field: 0.845, Grass land: 0.824, Forest: 0.847, and Wetland: 0.82. By comparison of the average NDVI of different seasons, the value of forest is greater than that of the crop field; the crop field is greater than grass land; grass land is greater than the paddy field, with the wetland being the lowest. The increasingly warm and dry conditions in the study region may cause vegetation cover to decline, Although vegetation in some areas increased, further investigation revealed that increases were mainly in croplands, which have been significantly influenced by irrigation. Another reason is the increase in extreme climate events such as droughts, heat waves, and rainstorms (Barriopedro et al. 2012), Liu et al. predicted an increase in drought events in Northeast China (Ke et al. 2012). In addition, the altering surface albedo and evapotranspiration also affect NDVI changes (Xu et al. 2012).

### Correlations between NDVI, temperature and rainfall

Climate changes is one of the most important factors driving vegetation growth. The close relationship between climate change and vegetation growth has been demonstrated in many previous studies (Piao et al. 2006). In this study, the spatial distribution of correlation between NDVI and climate factors showed that temperature dynamics slowly changed into rainfall dynamics from the upstream to the downstream areas. The regression model revealed that NDVI for Paddy field could be explained by positive correlation with rainfall and log rainfall; NDVI for crop field, forest and grassland could be explained by positive correlation with rainfall; and NDVI for wetland could be explained by positive correlation with Maxtemp. Rainfall in the region increased during the 12 years period and this climatic variable strongly impacted paddy field, crop field, forest and grassland. These land types are found mainly in the central and northwestern part of the region where temperatures are higher and the amount of rainfall are lower. Most areas displayed significant positive correlations between NDVI and rainfall (Duan et al. 2011; Chuai et al. 2013), While abundant rainfall is considered as an important factor for vegetation growth, but for wetlands, temperature serves as the priority. In contrast to the positive relationship between NDVI and precipitation, an inverse NDVI-temperature relationship was observed over most of the land use type. Although the warming trend should extend the growing season and potentially enhance vegetation growth (Fang et al. 2004; Peng et al. 2013; Nemani et al. 2003). However, this study analyzed only the correlation NDVI, temperature and rainfall, in addition it does not account for all vegetation variation. There are other factors that need to be considered on the influence of terrestrial vegetation growth, such as relative humidity, nutrients, light intensity and mechanical factors including wind and occurrence of fire, and so on (Breckle 2002). These need to be further studied.

## Conclusions

Vegetation coverage has been identified with the function of water and soil conservation and soil pollutants interception. Moreover, the interaction between NDVI and climate factors is obvious. Climate changes have been observed in the study area as precipitation decreased and temperature increased during the period of 2001–2010 as opposed to the same analysis in the early 1990s (Piao et al. 2006; Gao et al. 2009), when the results are just the opposite. This change in the climate proved to be a positive effect on vegetation coverage. The moderate increase of NDVI for forest and wetland showed the improvement of the upstream ecological system where water is conserved and water and soil erosion better are prevented. As China’s major grain production area, the region is rich in its land resources. The increase of NDVI for the paddy field and crop field also showed the expansion of crop coverage and increased grain yields. For grass land, although its NDVI is weakly increased, the problem of soil desertification is ameliorated due to its increased vegetation coverage and biomass. Quantitative measurement and spatial analysis are used to reveal the impact of temperature and rainfall on vegetation greenness and dynamics of various lands cover types in the region; and the results provide a better understanding of the fact that the vegetation greenness and the dynamics are responsive to climate change. These can be used to evaluate the impact of ecological restoration on vegetation types in the region in future.
